# Immune Dysregulation Mimicking Systemic Lupus Erythematosus in a Patient With Lysinuric Protein Intolerance: Case Report and Review of the Literature

**DOI:** 10.3389/fped.2021.673957

**Published:** 2021-05-20

**Authors:** Josefina Longeri Contreras, Mabel A. Ladino, Katherine Aránguiz, Gonzalo P. Mendez, Zeynep Coban-Akdemir, Bo Yuan, Richard A. Gibbs, Lindsay C. Burrage, James R. Lupski, Ivan K. Chinn, Tiphanie P. Vogel, Jordan S. Orange, M. Cecilia Poli

**Affiliations:** ^1^Facultad de Medicina Universidad del Desarrollo-Clínica Alemana, Santiago, Chile; ^2^Universidad de Chile, Reumatóloga Pediátrica Hospital San Juan de Dios, Santiago, Chile; ^3^Unidad de Inmunología y Reumatología Hospital Luis Calvo Mackenna, Providencia, Chile; ^4^Patológo Renal, Departamento de Anatomía Patológica, Facultad de Medicina, Pontificia Universidad Católica de Chile, Santiago, Chile; ^5^Department of Molecular and Human Genetics, Baylor College of Medicine, Houston, TX, United States; ^6^Human Genetics Center, Department of Epidemiology, Human Genetics, and Environmental Sciences, School of Public Health, The University of Texas Health Science Center at Houston, Houston, TX, United States; ^7^Department of Laboratories, Seattle Children's Hospital, Seattle, WA, United States; ^8^Department of Laboratory Medicine and Pathology, University of Washington, Seattle, WA, United States; ^9^Sequencing Center, Baylor College of Medicine, Houston, TX, United States; ^10^Texas Children's Hospital, Houston, TX, United States; ^11^Department of Pediatrics, Division of Allergy, Immunology and Retrovirology, Baylor College of Medicine, Houston, TX, United States; ^12^Department of Pediatrics, Division of Rheumatology, Baylor College of Medicine, Houston, TX, United States; ^13^Department of Pediatrics, Vagelos College of Physicians and Surgeons, New York Presbyterian Morgan Stanley Children's Hospital, Columbia University, New York, NY, United States; ^14^Unidad de Inmunología y Reumatología, Hospital Roberto del Río, Santiago, Chile

**Keywords:** lysinuric protein intolerance, systemic lupus erythematosus, hemophagocytic lymphohistiocytosis, immune mediated glomerulonephritis, case report, systemic lupus–erythematosus

## Abstract

Lysinuric protein intolerance (LPI) is an inborn error of metabolism caused by defective transport of cationic amino acids in epithelial cells of intestines, kidneys and other tissues as well as non-epithelial cells including macrophages. LPI is caused by biallelic, pathogenic variants in *SLC7A7*. The clinical phenotype of LPI includes failure to thrive and multi-system disease including hematologic, neurologic, pulmonary and renal manifestations. Individual presentations are extremely variable, often leading to misdiagnosis or delayed diagnosis. Here we describe a patient that clinically presented with immune dysregulation in the setting of early-onset systemic lupus erythematosus (SLE), including renal involvement, in whom an LPI diagnosis was suspected post-mortem based on exome sequencing analysis. A review of the literature was performed to provide an overview of the clinical spectrum and immune mechanisms involved in this disease. The precise mechanism by which ineffective amino acid transport triggers systemic inflammatory features is not yet understood. However, LPI should be considered in the differential diagnosis of early-onset SLE, particularly in the absence of response to immunosuppressive therapy.

## Introduction

Lysinuric protein intolerance (LPI) is an inborn error of metabolism characterized by the defective transport of cationic amino acids (CAA) arginine, lysine and ornithine through the basolateral membrane of the small intestine and renal tubular epithelial cells as well as in other non-epithelial cells such as monocytes/macrophages. Due to the poor intestinal uptake and loss of amino acids in the urine, patients have decreased plasma levels and increased urinary levels of CAA. A high incidence of this disease has been found in Finland (1:60000) (1, 2), Southern Italy (3), and Northern Japan (4), but cases have also been reported in Korea (5), Turkey (6), Malaysia (7), China (8), and Mexico (9).

LPI is caused by biallelic pathogenic variants in *SLC7A7* (10). *SLC7A7* maps to chromosome 14q11.2 (11), and encodes amino acid transporter 1 (y+LAT1). y+LAT is the light subunit isoform of the y+L system; it associates with the heavy subunit 4F2hc to form a heterodimeric transporter responsible for the transport of CAA, allowing the efflux of CAA from polar and non-polar cells (12). The molecular basis of LPI is limited to *SLC7A7* variants, as pathologic loss of *SLC3A2*, which encodes 4F2hc, has not been described in humans (13).

Clinical manifestations of LPI are widely variable, which often leads to a delayed or missed diagnosis (14–16). Symptoms may appear after weaning breastmilk and often resemble the findings in urea cycle disorders such as hyperammonemia, due to the depletion of key urea cycle intermediates. Hyperammonemia may explain other common LPI symptoms, including cognitive delay due to hyperammonemia that may sometimes be unrecognized, emesis, and aversion to protein-rich food. This urea cycle dysfunction is managed using a low protein diet, L-citrulline supplementation, and nitrogen-scavenging agents. Oral lysine has also been used in some patients but may not always be tolerated (17).

Systemic disease has also been reported with LPI, including pulmonary, neurologic, hematologic, renal and immune manifestations such as pulmonary alveolar proteinosis (PAP), hemophagocytic lymphohistiocytosis (HLH) and systemic lupus erythematosus (SLE). Other possible clinical manifestations include osteoporosis, glomerulonephritis, anemia and hepatosplenomegaly. These clinical manifestations cannot be explained exclusively by urea cycle dysfunction and are hypothesized to be related to primary immune dysfunction, although the molecular mechanism remains unclear (18).

We present the case of a 30-month-old child with LPI who presented with progressive renal inflammation suggesting SLE, in whom LPI diagnosis was suggested by exome sequencing (ES), followed by a literature review of the heterogenous manifestations of LPI and the current knowledge of the pathogenic mechanisms underlying this disease.

## Methods

### Exome Sequencing

Research exome sequencing (ES) was performed on the personal genomes of the proband and both parents who were computationally analyzed as a trio. Rare variants with a frequency of <0.1% were parsed and filtered; analysis was explored using different inheritance hypotheses including *de novo*, homozygous, heterozygous and compound heterozygous inheritance. Frequency, phenotype association and combined annotation dependent depletion (CADD), scores were considered for the analysis (For extended methodology see Supplementary Methods).

### Literature Review

A search was performed in PubMed using the following keywords: “lysinuric protein intolerance,” “clinical presentation,” “hemophagocytic lymphohistiocytosis,” “SLC7A7,” and “systemic lupus erythematosus.” Literature written in Spanish or English between 1990 and 2019 were reviewed. Aside from case reports, publications reporting more than one patient were also reviewed if the clinical presentations were sufficiently detailed. Publications that reported the same patient or group of patients were included if they detailed different multisystemic presentations (for example, renal vs. pulmonary presentations of the same group of patients). If more than one publication included the same patient(s), the publication containing more information was included. The total number of patients used for percentage calculation was determined with each patient counted once regardless of the number of publications in which a same patient appeared. Patients presenting simultaneously with pathogenic variants associated with other genetic diagnoses were excluded from the analysis. The program cBioPortal mutation mapper (https://www.cbioportal.org/mutation_mapper) was used to map the different variants.

## Case Report

A male patient, the second child of non-consanguineous parents, was born at 40 weeks with appropriate length and weight for his gestational age. The patient was exclusively breastfed until 6 months of life, when solids were gradually introduced while continuing breast milk until 2 years 5 months, after which he received formula. He was healthy until 12 months of life when he started to develop failure to thrive and presented with recurrent episodes of upper and lower respiratory tract infections, including multiple episodes of otitis media. He received immunizations according to the Chilean Immunization program until 12 months, but he did not receive vaccines corresponding to 18 months.

At age 2 years 6 months, the patient presented with multilobar pneumonia that required hospitalization. At this time, he showed persistent failure to thrive, below third percentile for height (z score: −2.07) and weight (z score −2.49). Viral testing was positive for rhinovirus.

He showed initial improvement with antibiotic therapy for presumed secondary bacterial pneumonia but required re-hospitalization due to respiratory deterioration and concomitant bilateral suppurative otitis media that required intravenous antibiotic therapy. Laboratory testing showed bicytopenia (hemoglobin = 9 g/dL, hematocrit = 28%, platelets = 84,000 cells/uL, white blood cells = 6,000 cells/uL) and elevated erythrocyte sedimentation rate (136 mm/hr). He developed generalized lymphadenopathy, hepatomegaly, hypoalbuminemia and elevated lactate dehydrogenase (718 IU/L, normal range 100–300 IU/L). A primary immune deficiency was suspected and immunoglobulin replacement was initiated (Table 1). The patient developed persistent fevers despite antibiotic treatment with ampicillin-sulbactam, cefotaxime, and clindamycin. Bronchoalveolar lavage was positive for *Mycoplasma pneumoniae*, and azithromycin was added.

**Table 1:** Patient's laboratory testing.

**Laboratory tests**	**Admission age 30 months**	**Age 31 months**	Age 32 months^*^
Hemoglobin (g/dL)	7.5 **↓**	8.2 ↓	3.9 ↓
Platelets (cells/uL)	278,000	76,000 ↓	58,000 ↓
Albumin (g/dL)	2.8 ↓	1.6 ↓	3.2 ↓
LDH (U/L)	718 ↑	2,263 ↑	1,669 ↑
BUN/Creatinine (mg/dL)	7/0.38	51/1.32	40/0.99
Amylase/Lipase (U/L)	–	211/6,155	103/11,085
IgG-IgA-IgM-IgE (mg/dL)	–	2205-96-10-102	–
C3/C4 (mg/dL)	60 ↓/6.3 ↓	50 ↓/ 3.6 ↓	–
Total leukocytes (cells/uL)	10,120	5,370	3,800
CD3^+^ T cells/mm^3^	1,714 (normal 900–4,500)	–	–
CD4^+^ T cells/mm^3^	659 (normal 500–2,400)	–	–
CD8^+^ T cells/mm^3^	758 (normal 300–1,600)	–	–
CD19^+^ B cells/mm^3^	35 (normal 200–2,100) ↓	–	–
NK cells	254 (normal 100–1,000)	–	–
Ferritin (ng/mL)	24	374 ↑	–
Fibrinogen (mg/dL)	161	577	342

* *An underlying metabolic disease was not suspected and ammonia was not determined during the course of the illness*.

The patient remained febrile and developed a generalized macular rash, periorbital edema and oliguria. Laboratory testing showed nephrotic range proteinuria (protein/creatinine ratio 14.4), hypoalbuminemia and bicytopenia (anemia and thrombocytopenia). He then developed complex partial seizures. In the context of acute progressive renal dysfunction with hemolytic anemia, a thrombotic microangiopathy was suspected and treated with high dose pulse steroids (30 mg/kg), five cycles of plasmapheresis and two doses of eculizumab. Due to severe renal dysfunction, the patient was placed on continuous dialysis and a renal biopsy was performed.

**Figure 1:**
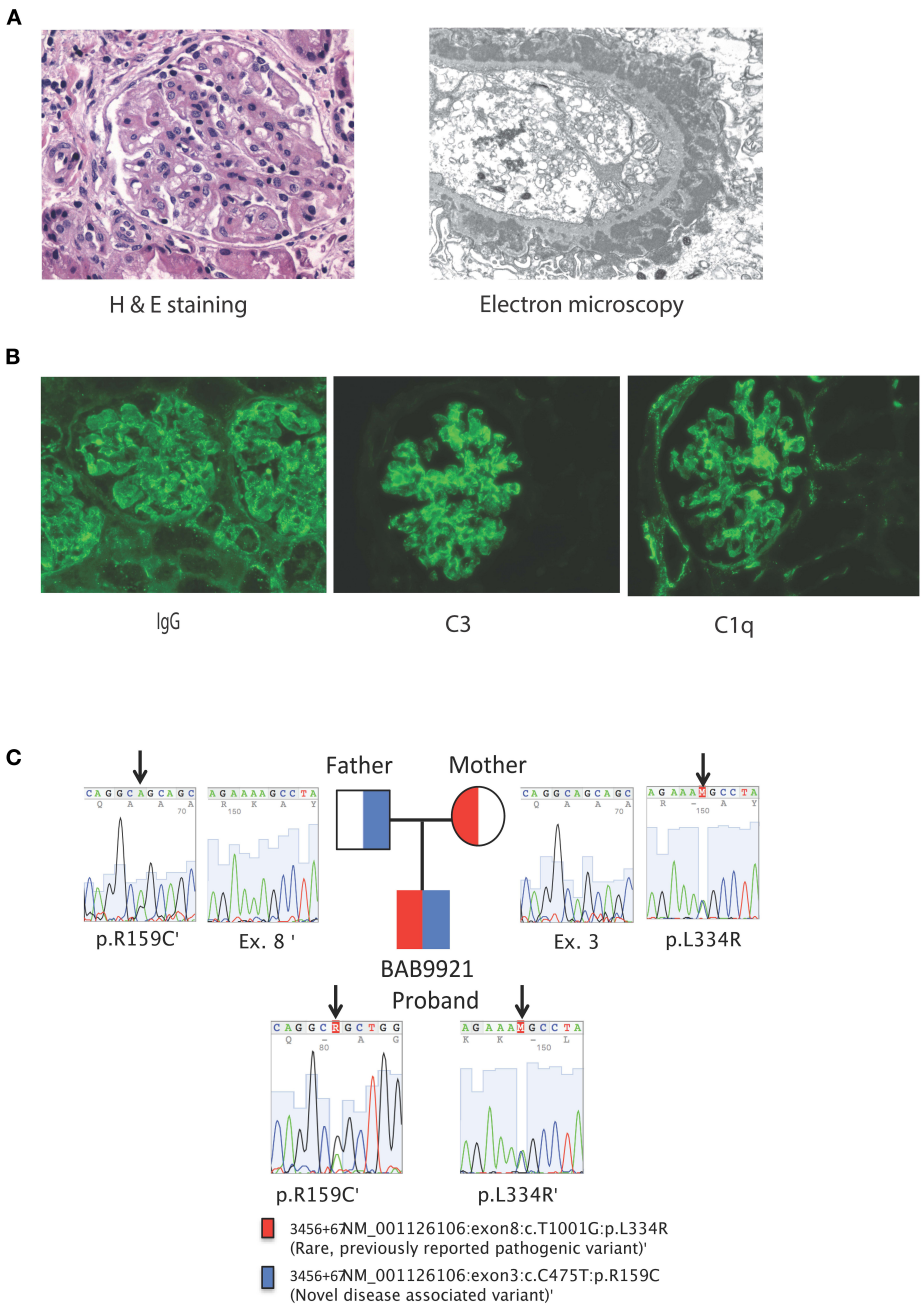
**(A)** From left to right, Kidney biopsy hematoxylin and eosin stain, original magnification x 400. The glomerulus shows a lobular architecture and capillary loops with increased thickness and double contours. There is endocapillary and mesangial proliferation with mononuclear cells. On the left, an arteriole shows moderate sclerosis. Kidney biopsy electron microscopy, uranyl acetate-lead citrate stain, original magnification x 6000. The glomerular capillary loop shows massive, confluent, subepithelial electron-dense deposits with spike formation from the basement membrane mimicking stage II of a membranous pattern of injury. There are several scattered, small electron-dense deposits in subendothelial areas. The endothelium is very swollen and contains multiple tubulo-reticular inclusions. **(B)** Kidney biopsy immunofluorescence with antibodies against IgG, C3 and C1q original magnification x 200. Both glomeruli show coarse and fine granular deposits in the mesangium and peripheral capillary loops. There are also some granular deposits at the tubular basement membranes. Immunofluorescence studies were also positive for C3 and C1q granular deposits, mainly at the mesangium, but also present on segments in a subendothelial location. **(C)** Family pedigree and Sanger tracings of patient *SLC7A7* variants.

Rheumatologic evaluation showed an elevated ANA titer with a speckled pattern; Smith, and SSA, antibodies were also positive, but anti-double stranded DNA testing was inconclusive (Table 2). Kidney biopsy showed immune complex deposition consistent with early-onset SLE, therefore, intravenous cyclophosphamide was initiated. Despite these immunosuppressive interventions, the patient had a severe and progressive course with pancytopenia, hyperferritinemia and elevated triglycerides suggesting macrophage activation syndrome (MAS). Simultaneously, he developed elevated pancreatic enzymes consistent with acute pancreatitis (amylase = 211 U/L, normal <20; lipase = 6,155 U/L, normal <25). In this setting, he received three additional pulses of methylprednisolone and IVIG at an immunomodulatory dose (2 g/kg). Despite these interventions, the patient required continuous dialysis. He developed septic shock and disseminated intravascular coagulation with multisystem failure, leading ultimately to his death 45 days after admission. 3 days postmortem, the final renal biopsy report demonstrated an immune complex-mediated glomerulonephritis (Figures 1A,B).

**Table 2:** Auto-antibody testing.

**Autoantibodies**	**Admission age 30 months**
Anti-nuclear	1:320 (+)
SSA	81 (+)
SSB	6 (–)
Smith	72 (+)
Scl-70	2 (–)
Jo-1	3 (–)
PR3/MPO	Negative

**Figure 2:**
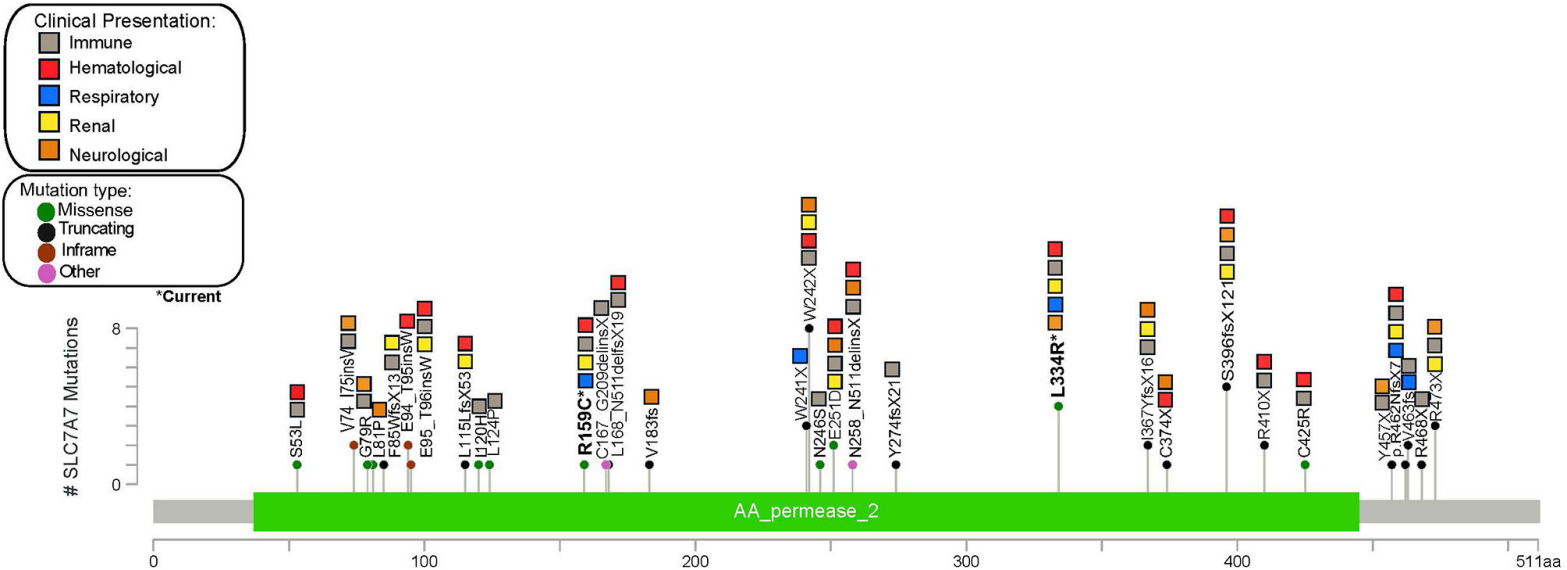
Gene map showing the frequency and clinical manifestations associated with different *SLCA7A* variants described in the reviewed literature.

A previously collected blood sample from the proband was sent after his demise along with parental samples for research genetic studies by trio ES which showed compound heterozygous missense variants in *SLC7A7; a novel* NM_001126106:c.475C>T (p.Arg159Cys) variant in exon 3 with a CADD score of 28.4 and a known pathogenic variant NM_001126106:c.1001T>G (p.Leu334Arg) with a CADD score of 33 suggesting a molecular diagnosis of LPI. There were no other rare variants identified on ES that could explain the patient's phenotype, in particular no variants associated with hemophagocytic lymphohistiocytosis (HLH) were observed. The c.475C>T (Arg159Cys) variant has not been reported in patients to-date. Thirty individuals from the gnomAD database (https://gnomad.broadinstitute.org) were heterozygous carriers for this variant, while no homozygote was observed. ClinVar documented three conflicting classifications for this variant; likely pathogenic, variant of uncertain significance (VUS), and Likely benign (https://www.ncbi.nlm.nih.gov/clinvar/variation/203ro944/). We classified the c.475C>T (Arg159Cys) variant in *SLC7A7* as a variant of uncertain significance according to the guidelines provided by American College of Medical Genetics and Genomics (19). ES also showed that both parents were carriers for *SLC7A7* variants; the mother was heterozygous for Leu334Arg and the father was heterozygous for Arg159Cys variants respectively, in accordance with Mendelian expectations for an autosomal recessive disease trait (Figure 2).

## Results

A total of 62 publications with 157 patients with LPI were found (Table 3). The incidence of metabolic and gastrointestinal symptoms corresponded to 52% (*n* = 82) and 43% (*n* = 68), respectively, while hematologic (55%, *n* = 86), immune (54%, *n* = 85) and renal (41%, *n* = 65) manifestations were also prevalent. The main metabolic and gastrointestinal symptoms were failure to thrive (52%), protein-rich food aversion (36%), and emesis (30%), while the main multisystemic manifestations included hepatosplenomegaly (46%), proteinuria (34%), anemia (29%), and osteoporosis (24%). With regards to pulmonary (18%, *n* = 29) and neurologic (25%, *n* = 40) involvement, symptoms most frequently found were pulmonary alveolar proteinosis (PAP) (16%) and cognitive delay (11%). Five asymptomatic patients were described, however, one of them had postprandial hyperammonemia after a protein loading test, while another died suddenly of unknown causes. Five patients presented simultaneously with SLE and LPI, and three patients had lupus-like symptoms that were ultimately attributed to LPI (53–55). LPI was also reported to mimic other immune dysregulatory diseases including HLH (14, 22) and celiac disease (16), leading to initial misdiagnoses. An individual case of LPI has been reported with anti-N-methyl-D-aspartate receptor encephalitis (67).

**Table 3:** Clinical presentation of published LPI patients.

**Clinical presentation**	**Percentage % of patients**	**Notes**	**References**
Failure to thrive	52% (81/157)	Patients (20) and (21) were also diagnosed with growth hormone deficiency.	(3, 5–9, 14, 20–36)
Metabolic acidosis	6% (9/157)		(20, 26, 28, 30, 37)
Emesis	30% (47/157)	(14) Both patients developed emesis during their 1st year of life.	(3, 6, 8, 14–16, 21, 25, 27, 34, 35, 38–45).
Diarrhea	14.65% (23/157)		(5, 14–16, 21, 22, 27, 32, 34, 42, 43)
Protein-rich food aversion	36% (56/157)	(35) Patient was initially reported as not having proteinrich food aversion, but developed it after being submitted to a fructose free diet.	(3, 6–8, 15, 16, 20, 24, 25, 27, 32, 35, 38, 44, 46–53)
Osteoporosis	24% (38/157)		(3, 6, 8, 24, 28, 29, 34, 44, 48, 50, 52)
Osteopenia	13% (21/157)	(54) One patient also had recurrent fractures.	(16, 27, 38, 40, 54)
Muscular hypotonia	16% (25/157)		(3, 9, 14, 22, 24, 34, 55–58)
Pulmonary alveolar proteinosis (PAP)	16% (25/157)		(3, 8, 9, 37, 54, 59–61)
Pneumonia	6% (10/157)	(41) Patient had three hospitalizations due to pneumonia and was diagnosed a fourth time on admission. (47) Two patients had pneumonia secondary to varicella.	(8, 9, 41, 47, 49, 59)
Proteinuria	34% (53/157)		(3, 6, 22, 27, 28, 30, 31, 48, 49, 58, 62)
Tubulopathies	14% (22/151)	Patients (26) and (3) had Fanconi syndrome. Three patients reported in (28) had chronic tubulointerstitial nephritis and another had slight mesangial thickening.	(3, 20, 26–28, 31)
Hematuria	16% (25/157)		(22, 28, 30, 31, 63)
Glomerulonephritis	4% (7/157)		(30, 37, 48)
Pancreatitis	4% (7/157)		(3, 27, 30, 49)
Hepatosplenomegaly	46% (72/157)	Only hepatomegaly: 2% (18/151) Only splenomegaly: 1.3% (2/151)	(3, 5, 7, 8, 14, 20, 24, 28, 30–32, 34, 37–39, 42, 43, 47, 49, 51, 55, 58, 63–66)
Fever	9% (14/157)		(26, 30, 36, 37, 54, 63, 66, 67)
Recurrent bacterial or viral infections	7% (11/157)	Infections were all located in the respiratory track, except for (59), who had recurrent urinary tract infections.	(8, 30, 36, 38, 41, 42, 59, 68)
Leukopenia	11% (17/157)	Only lymphopenia: (53) Neutropenia and lymphopenia: (22) Only neutropenia: (54)	(5, 14, 22, 23, 53, 54, 65)
Hemophagocytic lymphohistiocytosis (HLH)	14% (22/157)		(14, 21, 25, 27, 54, 55)
Systemic lupus erythematosus (SLE)	3% (5/157)	Patient (27) presented with lupus nephritis.	(27, 48, 49, 57, 63)
Other or non-specified immune abnormality	8% (12/157)	(54) Three patients had autoimmunity. One presented with SLE-like symptoms while another had a rheumatoid arthritis like presentation. In (27) one patient had vitiligo and another had immune thrombocytopenic purpura. Rheumatoid arthritis (RA): (57) Anti-NMDAR encephalitis: (67)	(9, 27, 47, 54, 57, 67)
Lethargy or stupor	9% (14/157)		(3, 5, 26, 38, 44, 46, 50, 53)
Loss of consciousness or coma	9% (14/157)	In (32), coma was the presenting symptom of LPI.	(3, 27, 28, 37, 38, 44, 46)
Seizures	8% (12/157)		(3, 21, 27, 46, 54, 67)
Cognitive delay	11% (17/157)	Mild to moderate mental retardation reported.	(3, 6, 7, 24, 26, 28, 69, 70)
Hyperlipidemia	16% (25/157)	A total of nineteen patients had hypertriglyceridemia (6, 14, 22, 27, 36, 54), five had combined hyperlipidemia (6, 23, 59, 62), and one had hypercholesterolemia (6).	(6, 14, 22, 23, 27, 54)
Anemia	29% (45/157)		(5, 7, 14, 24, 27–29, 35, 39–41, 43, 49, 53–56)
Thrombocytopenia	18% (28/157)		(14, 22, 31, 48, 49, 53–55, 55, 56, 71)
Coagulopathy	15% (24/157)	(54) Patients had hypofibrinogenemia.	(14, 22, 23, 27, 36, 48, 49, 54, 61, 66)
Hypertension	14% (22/157)		(28, 32, 57, 62)
Asymptomatic	3% (5/157)	(63) Although patient was asymptomatic, postprandial hyperammonemia was observed after protein loading test. (33) Patient suffered a sudden death.	(7, 31, 33, 63, 72)

Overall, several clinical manifestations of LPI where immune-related and these include positivity to various autoantibodies in six patients (4%) and SLE-like disease in 3% of reviewed cases including this report, all of whom showed positive ANA autoantibodies among others that were also present (23, 46, 55, 64, 73) while immune complex mediated glomerulonephritis was seen in 4% of patients. Cytopenias were also a frequent finding with anemia being the most common finding in 29% of patients, leucopenia was seen in 11% and thrombocytopenia in 18%. MAS/HLH which is probably the most severe expression of immune dysregulation was reported in 22 patients (14%) including the current case. PAP which is most likely also immune- mediated, was seen in 25 (16%) of the reviewed cases. Single cases of other autoimmune disorders including vitiligo (74, 75), immune thrombocytopenic purpura (74) and rheumatoid arthritis (76) were also identified.

Three pregnant patients were also reported (23, 46, 64). Symptoms appearing during pregnancy consisted mainly of anemia and thrombocytopenia, although proteinuria was observed in one case. Pregnancy in women with LPI can be associated with increased risk of complications including hyperammonemia, preeclampsia, intrauterine growth restriction and post-partum hemorrhage requiring close monitoring (64).

Of 62 publications, 27 reported the patients' variants, totaling 61 individuals with known pathogenic variants identified. A total of 39 different *SLC7A7* pathogenic variants were reported, most of which were located in exon 3 (31%, *n* = 12), followed by exon 5 (13%, *n* = 5) and exon 10 (13%, *n* = 5). The most frequent pathogenic variant reported is p.W242^*^, found in eight patients, followed by p.S396fs^*^12 and p.L334R (present in our current case), which have been identified in five and four other patients, respectively (Figure 1). Through this review we were unable to identify a specific genetic hotspot. Interestingly, however, cytosine to thymidine transitions were slightly overrepresented (6 out of 21 SNVs), suggesting the presence of susceptible CpG sites (73). Point mutations were most frequently seen, corresponding to a total of 19 patients with non-sense variants and 15 with missense variants. Large deletions have also been reported and should be considered in patients with suggestive clinical features and negative sequencing findings (24, 29). No correlation between clinical presentation and variant location was found from our review, as the broad clinical spectrum was homogeneously distributed among patients with different variants throughout the gene. Furthermore, patients presenting with the same homozygous variant differed in symptoms and type of systemic involvement.

## Discussion

Here we report the case of a patient with suspected diagnosis of LPI and a review of the literature including recent LPI case reports. Interestingly our patient presented with lupuslike disease, including lupus nephritis. Since LPI diagnosis was not suspected during his clinical course, ammonia, plasma and urine amino acids were never assessed. Diagnosis was suspected post-mortem after trio ES analysis revealed compound heterozygous variants in *SLC7A7*, considering our patient had presented with multiple clinical features that were reminiscent of LPI including, failure to thrive, recurrent infections and overwhelming inflammation in the context of infection with macrophage activation and renal failure. It is important to note however, that this patient carried one known *SLC7A7* pathogenic variant and a VUS for which there is not enough bioinformatic, clinical and functional evidence to claim pathogenicity. Unfortunately, further testing is not possible given that the patient is deceased. This case highlights the need to consider LPI in the differential diagnosis of patients with similar constellation of symptoms to pursue appropriate testing that enables one to confirm or rule out the diagnosis and initiate therapy targeting the urea cycle dysfunction. Literature review supported the fact that individuals with LPI can present with a wide spectrum of multisystemic manifestations, revealing some features to be almost as common as the anticipated metabolic and gastrointestinal symptoms. Unexpectedly, gastrointestinal and metabolic symptoms were not reported in all cases and this may be due to the nature of information given in the case reports/series. Some publications included large groups of patients focused mainly on novel multisystemic symptoms of LPI and did not highlight failure to thrive or emesis among the clinical manifestations of these patients, likely leading to underestimation of patients displaying these types of symptoms. In addition, a genotype-phenotype relationship could not be established, eliminating the possibility of determining disease severity and clinical presentation exclusively with genetic studies, which remain as the key tool for diagnosis or confirmation of LPI.

Immune dysfunction is a well-known complication of LPI, although the mechanism by which LPI generates this dysfunction remain unclear. CAA transport, especially of arginine, in non-polarized (epithelial) cells such as macrophages and lymphocytes is mainly carried out by y+LAT1 which decreased in patients with LPI (74, 75). Thus, it has been hypothesized that impaired arginine efflux results in increased intracellular arginine concentrations and augments nitric oxide (NO) production (12). Arginine is the exclusive substrate of inducible nitric oxide synthase (iNOS) which catalyzes the conversion of L-arginine to L-citrulline and NO. Augmented availability of arginine could cause hyperproduction of NO in LPI, which could result in CD8^+^T lymphocyte activation, excessive cytokine production and ectopic migration of leukocytes leading to the immune phenotypes seen in LPI patients, such as hepatosplenomegaly, HLH and autoimmunity (18, 76). This idea is supported in a study by Manucci et al. (77), who reported increased nitrates in LPI patient plasma and nitrites in LPI patients fibroblasts, accompanied by an increase of plasma levels of L-citrulline, suggesting iNOS activity.

However, an alternative pathogenic model was presented by Barilli et al. (75), where monocytes with mutated y+LAT1 induced an inflammatory phenotype in individuals with LPI through an arginine independent pathway. Downregulation of y+LAT1 in monocytes directly produced increased cytokines, that acted on hyperreactive epithelial airway cells to recruit more defective monocytes, thus entering into a feedback loop amplification. This phenomenon could be replicated in other systems in the body, explaining the multisystemic manifestations and suggesting that LPI may be an autoinflammatory disease. They proposed that this model could explain the high incidence of HLH and SLE-like symptoms in LPI. In addition, deficient toll-like receptor function in LPI macrophages has also been reported, which could also contribute to immune alterations.

Immune dysfunction could also be associated with other systemic manifestations of LPI, including pulmonary and immune-mediated renal failure. Common pulmonary manifestations include recurrent respiratory tract infections, pneumonia and PAP. PAP is largely attributed to deficient surfactant clearance, in which alveolar macrophages (AM) play a large role. Disruption of normal surfactant homeostasis due to AM dysfunction leads to the accumulation of this substance, therefore triggering the disease. This issue is highlighted in a case reported by Santamaria et al. (68). A patient with LPI experienced recurrent PAP, with recurrent disease even after a lung-heart transplantation, which suggested colonization by defective circulating monocytes/macrophages that could be responsible for the condition. Chronic renal injury could also be generated by inflammatory processes secondary to dysregulated immune activation caused by excess NO production (78). Efferocytosis is the process by which macrophages and other phagocytic cells remove apoptotic cells; recently Demy et al. showed that y+LAT1 is expressed shortly after efferocytosis in microglia and other tissue resident macrophages allowing them to preserve their own viability during phagocytosis, defects in macrophage phagocytic function could contribute to both neurologic and immunologic finding in patients with LPI (79).

Standard treatment in LPI includes a protein-restricted diet, nitrogen-scavenging agents, and L-citrulline administration to replenish the urea cycle, which reduce risk for hyperammonemia (12). In addition, L-carnitine and L-lysine may be recommended in particular circumstances, although lysine as a long-term treatment is sometimes avoided due to the incidence of gastrointestinal intolerance (12). Management of the immunological phenotypes of LPI is still under research and, while replenishment of the urea cycle may help, it is unclear how arginine-independent pathways of macrophage activation may be targeted. Until recently, the lack of animal models has hindered the process of determining specific treatment targets. A recently reported mouse model recapitulates LPI at a biochemical and phenotypic level constituting a promising subject for future studies focused on therapeutics (80), although it is limited by poor survival. An inducible *SLC7A7* knockout mouse model showed higher viability and similarities to human LPI (81).

## Conclusions

LPI is a severe metabolic disorder that can present with a wide range of systemic features, including immune dysregulation. Impaired lymphocyte function, hypocomplementemia, immune-mediated glomerulonephritis, autoantibodies, and HLH are known complications of LPI, although exactly how ineffective amino acid transport triggers these systemic inflammatory features is not yet understood. Furthermore, no clear genotype-phenotype correlation exists in patients with LPI, making it difficult to predict disease severity and presentation. Due to the similarities in clinical presentation, LPI should be considered in the differential diagnosis of early-onset SLE, particularly in the absence of response to immunosuppressive therapy.

## Data Availability Statement

The datasets generated for this study can be found in online repositories. The names of the repository/repositories and accession number(s) can be found below: phs000711.v6.p2, phs000711.v7.p2.

## Ethics Statement

The studies involving human participants were reviewed and approved by Baylor College of Medicine Institutional Review Board. Written informed consent to participate in this study was provided by the participants' legal guardian/next of kin.

## Author Contributions

JC and MP wrote the manuscript. ML and KA were the main physicians in charge of in the patient's clinical care. GM analyzed and described pathology results of kidney biopsies. ZC-A, and IC performed and analyzed ES results. JRL and RG supervised data interpretation of genetic and genomic studies. MP supervised all aspects of this study. TV, JRL, and JO were involved in study design and critically revised the manuscript. LB critically revised the manuscript and provided expert feedback. All authors reviewed and approved the manuscript.

## Conflict of Interest

The authors declare that the research was conducted in the absence of any commercial or financial relationships that could be construed as a potential conflict of interest.
