# Supplementary material for: Immune Dysregulation Mimicking Systemic Lupus Erythematosus in a Patient With Lysinuric Protein Intolerance: Case Report and Review of the Literature
Source: Front Pediatr. 2021 May 20;9:673957. doi: 10.3389/fped.2021.673957 (PMC8172984; doi:10.3389/fped.2021.673957)
Supplement: Supplementary file 1 [file Table_1.DOCX]

| **Gene(s)** | **Chromosomal change (hg19)** | **Reference sequence and effect** | **Zygosity** | **vR / tR** | **Inheritance** | **Mutation type** | **gnomAD^a^** | **CADD^b^** | **pLI^c^** | **pRec^d^** |
| --- | --- | --- | --- | --- | --- | --- | --- | --- | --- | --- |
| Recessive mode analysis results | | | | | | | | | | |
| *FLNA* | X:153577856_C>A | NM_001456  c.7606G>T:p.A2536S | Hemi | 76 / 77 | Mat | Nonsynonymous | 0 | 0.018 | 0.9999 | 1.24x10^8^ |
| *HCFC1* | X:153215873_G>T | NM_005334  c.5825C>A:p.T1942N | Hemi | 153 / 154 | Mat | Nonsynonymous | 0 | 13.62 | 0.9999 | 4.07x10^6^ |
| *BCLAF3* | X:19984386_A>T | NM_198279  c.423T>A:p.D141E | Hemi | 46 / 46 | Mat | Nonsynonymous | 0 | 11.22 | N/A | N/A |
| *DDX53* | X:23019879_A>T | NM_182699  c.1705A>T:p.T569S | Hemi | 63 / 63 | Mat | Nonsynonymous | 0 | 11.71 | 0.8254 | 0.1738 |
| *IRS4* | X:107978929_C>T | NM_003604  c.646G>A:p.E216K | Hemi | 52 / 53 | Mat | Nonsynonymous | 0 | 12.27 | 0.6094 | 0.3902 |
| *PLXNB3* | X:153037386_G>A | NM_001163257  c.2654G>A:p.R885Q | Hemi | 129 / 129 | Mat | Nonsynonymous | 0.0000171 | 6.964 | 0.1033 | 0.8967 |
| *ZFY* | Y:2847262_T>C | NM_001145276  c.1061T>C:p.I354T | Hemi | 41 / 41 | Pat | Nonsynonymous | 0 | 19.26 | 0.2555 | 0.6405 |
| *USP17L4* | 8:7194973_CA>C | NM_001256874  c.338delA:p.H113fs | Het | 33 / 140 | Mat | Frameshift deletion | 0 | N/A | N/A | N/A |
|  | 8:7195818_TG>T | NM_001256874  c.1183delG:p.G395fs | Het | 41 / 89 | Pat | Frameshift deletion | 0.000129 | N/A |  |  |
| *AHNAK2* | 14:105418729_A>G | NM_138420  c.3059T>C:p.L1020S | Het | 10 / 93 | DN | Nonsynonymous | 0.000298 | 0.004 | N/A | N/A |
|  | 14:105410483_C>T | NM_138420  c.11305G>A:p.V3769M | Het | 34 / 137 | Mat | Nonsynonymous | 0.0000763 | 13.91 |  |  |
| *FER1L5* | 2:97334624_C>A | NM_001113382  c.1135C>A:p.R379S | Het | 44 / 79 | Mat | Nonsynonymous | 0.000026 | 3.659 | N/A | N/A |
|  | 2:97334836_G>A | NM_001113382  c.1309G>A:p.G437S | Het | 43 / 77 | Pat | Nonsynonymous | 0.00000649 | 16.84 |  |  |
| *FLNC* | 7:128497246_T>A | NM_001458  c.7636T>A:p.S2546T | Het | 44 / 420 | DN | Nonsynonymous | 0 | 24 | 0.9995 | 0.0005 |
|  | 7:128497219_G>A | NM_001458  c.7609G>A:p.A2537T | Het | 64 / 453 | Pat | Nonsynonymous | 0.00126 | 14.52 |  |  |
| *SLC7A7* | 14:23244747_A>C | NM_001126106  c.1001T>G:p.L334R | Het | 78 / 148 | Mat | Nonsynonymous | 0.00000796 | 28.4 | 0.0021 | 0.9780 |
|  | 14:23282133_G>A | NM_001126106  c.475C>T:p.R159C | Het | 163 / 346 | Pat | Nonsynonymous | 0.000107 | 33 |  |  |
| Dominant mode analysis results | | | | | | | | | | |
| *ANKS6* | 9:101513382_C>T | NM_173551  c.2327-4G>A | Het | 14 / 37 | DN | Splicing | 0 | N/A | 0.0003 | 0.9965 |
| *CRTC1* | 19:18879466_G>T | NM_015321  c.1183G>T:p.G395C | Het | 58 / 138 | DN | Nonsynonymous | 0 | 23.6 | 0.9806 | 0.0195 |
| *ENAH* | 1:225707069_C>A | NM_018212  c.633G>T:p.E211D | Het | 9 / 33 | DN | Nonsynonymous | 0 | 20.1 | 0.9957 | 0.0043 |
| *NPIPB5* | 16:22546279_C>T | NM_001135865  c.1975C>T:p.R659C | Het | 17 / 88 | DN | Nonsynonymous | 0 | 24.4 | N/A | N/A |

Supplemental Table 1: Variants identified in the patient.

^a^ Composite allelic frequency in the Genome Aggregation Database 2.1.1

^b^ Combined Annotation Dependent Depletion Phred score

^c^ gnomAD 2.1.1 probability of loss of function intolerance

^d^ gnomAD 2.1.1 probability of intolerance of homozygous but not heterozygous loss of function variants

Abbreviations: DN, *de novo*; Hemi, hemizygous; Het, heterozygous; Mat, maternal; md, potential disease modifier; N/A, not available; Pat, paternal; tR, total reads; vR, variant reads

**Supplemental Methods:**

*Exome sequencing*

Exome sequencing was performed on isolated genomic DNA from blood of the proband and both parents by the Human Genome Sequencing Center (HGSC) at Baylor College of Medicine through the Baylor-Hopkins Center for Mendelian Genomics (CMG) initiative. With 500 ng of genomic DNA, an Illumina paired-end pre-capture library was constructed according to the manufacturer’s protocol (Illumina) with modifications as described in the BCM-HGSC Illumina Barcoded Paired-End Capture Library Preparation protocol. Pre-capture libraries were pooled into 6-plex library pools used the custom VCRome 2.1^1^ capture reagent (42 Mb, NimbleGen) according to the manufacturer’s protocol (NimbleGen SeqCap EZ Exome Library SR User’s Guide) with minor revisions. The sequencing run was performed in paired-end mode using the Illumina HiSeq 2000 platform, with sequencing-by-synthesis reactions extended for 101 cycles from each end and an additional 7 cycles for the index read. With an average sequencing yield of 8.2 Gb and average coverage of 101x, samples achieved 97% of the targeted exome bases covered to a depth of 20X or greater. Illumina sequence analysis was performed using the HGSC Mercury analysis pipeline^2,3^ (<https://www.hgsc.bcm.edu/software/mercury>), also available via DNANexus (<http://blog.dnanexus.com/2013-10-22-run-mercury-variant-calling-pipeline/>), which moves data through various analysis tools from the initial sequence generation on the instrument to annotated variant calls (SNPs and intra-read in/dels). In parallel to the exome workflow an Illumina array was generated for a final quality assessment. This included orthogonal confirmation of sample identity and purity using the Error Rate In Sequencing (ERIS) pipeline developed at the HGSC. Using an “e-GenoTyping” approach, ERIS screens all sequence reads for exact matches to probe sequences defined by the variant and position of interest. A successfully sequenced sample must meet quality control metrics of ERIS SNP array concordance (>90%) and ERIS average contamination rate (<5%).

The ATLAS2 variant calling method and Sequence Alignment/Map were used to call variants, followed by a customized Cassandra annotation pipeline, which is based on Annotation of Genetic Variants. Raw sequencing data were parsed for rare variants using bioinformatics algorithms set to specific variant allele frequency controls. Insertions/deletions (indels) had to pass through quality controls and were parsed for minor allele frequency (MAF) <2% using the Thousand Genomes Project (1000GP) database to be reported^4^. However, indels reported as variants in the Human Gene Mutation Database only had to have an MAF <5% in 1000GP to be retained^5^. Single nucleotide variants also had to pass quality controls and were parsed for rare variants in 1000GP, National Heart Lung and Blood Institute GO Exome Sequencing Project (esp5400) African, and esp5400 European databases for initial inclusion.

Coding variants (frameshift, stopgain, stoploss, splicing, nonsynonymous, and indel) were then filtered using recessive and dominant mode analyses. Variants with quality scores less than 10 (maximum = 60) or for which the variant read:total read percentage did not reach at least 10% were removed. For recessive mode analyses, both autosomal recessive and hemizygous models were considered. Prospective autosomal recessive variants were excluded with an MAF greater than or equal to 0.5% in the following databases: internal CMG, gnomAD, ESP5400, and 1000GP. In the hemizygous model, variants with an MAF greater than or equal to 1% in the same databases were excluded. Compound heterozygous variants were disqualified if present within the same parent. Autosomal recessive variants with greater than 1 homozygous case in gnomAD were excluded. Similarly, hemizygous variants with more than 1 hemizygous or homozygous case in gnomAD were eliminated. For dominant mode analyses, variants were excluded if present in either parent. Heterozygous variants were further removed if present in internal CMG, ESP5400, or 1000GP databases at an MAF greater than or equal to 0.1% or if present in the gnomAD database in more than 1 case.

1. Bainbridge MN, Wang M, Wu Y, Newsham I, Muzny DM, Jefferies JL, Albert TJ, Burgess DL, Gibbs RA. Targeted enrichment beyond the consensus coding DNA sequence exome reveals exons with higher variant densities. Genome Biol. 2011. 12(7):R68. PubMed PMID: 21787409; PubMed Central PMCID: PMC3218830.
2. Challis D, Yu J, Evani US, Jackson AR, Paithankar S, Coarfa C, Milosavljevic A, Gibbs RA, Yu F. (2012) An integrative variant analysis suite for whole exome next-generation sequencing data.  BMC Bioinformatics. 2012 Jan 12;13:8. doi: 10.1186/1471-2105-13-8.
3. Reid JG, Carroll A, Veeraraghavan N, Dahdouli M, Sundquist A, English A, Bainbridge M, White S, Salerno W, Buhay C, Yu F, Muzny D, Daly R, Duyk G, Gibbs RA, Boerwinkle E. (2014) Launching genomics into the cloud: deployment of Mercury, a next generation sequence analysis pipeline.  BMC Bioinformatics. 2014 Jan 29;15:30. doi: 10.1186/1471-2105-15-30.
4. Abecasis GR, Altshuler D, Auton A, Brooks LD, Durbin RM, Gibbs RA, Hurles ME, McVean GA, Donnelly P, Egholm M, Flicek P, Gabriel SB, Gibbs RA, Knoppers BM; 1000 Genomes Project Consortium. A map of human genome variation from population-scale sequencing. Nature. 2010;467(7319):1061–1073.
5. Stenson PD, Mort M, Ball EV, Evans K, Hayden M, Heywood S, Hussain M, Phillips AD, Cooper DN. The Human Gene Mutation Database: towards a comprehensive repository of inherited mutation data for medical research, genetic diagnosis and next-generation sequencing studies. Hum Genet. 2017;136(6):665–677.
